# Dual-Task Training Improves Shoulder Function and is Associated with Changes in Sensorimotor Network Connectivity After Arthroscopic Rotator Cuff Repair: A Randomized Controlled Trial

**DOI:** 10.1186/s40798-026-01069-y

**Published:** 2026-07-13

**Authors:** Liang Chen, Zi-Hao Jia, Peng Chen

**Affiliations:** 1 Department of Sports Medicine, The First Affiliated Hospital of Fujian Medical University, Fuzhou, 350005 Fujian China; 2 Department of Fujian Provincial Key Laboratory of Medical Instrument and Pharmaceutical Technology, Fuzhou University, Fuzhou, 350108 Fujian China; 3 College of Electrical Engineering and Automation, Fuzhou University, Fuzhou, 350108 Fujian China

**Keywords:** Arthroscopic rotator cuff repair, Dual-task training, Shoulder function, Brain functional connectivity, Randomized controlled trial

## Abstract

**Background:**

Recovery after arthroscopic rotator cuff repair (ARCR) is influenced by pain, proprioception, sensorimotor control, and psychological factors in addition to tissue healing. Cognitive–motor dual-task training may improve attentional allocation and motor control, but its role in postoperative ARCR rehabilitation remains unclear.

**Methods:**

In this randomized, parallel-group, controlled trial (Department of Sports Medicine, The First Affiliated Hospital of Fujian Medical University, China; January 2025–January 2026), 72 patients after ARCR were randomly assigned to a control group or a dual-task group (*n* = 36 each). Both groups received conventional rehabilitation, while the dual-task group additionally performed a serial subtraction-by-3 task during motor training. The intervention started at postoperative week 6 and lasted 12 weeks, with two supervised sessions per week. Assessments were conducted at postoperative weeks 6 and 18. Primary outcomes were the Constant–Murley Score (CMS) and the β-band weighted Phase Lag Index Network-Based Statistic difference (β-ΔwPLINBS). Secondary outcomes included the Visual Analog Scale (VAS), Absolute Angular Error (AAE), Disabilities of the Arm, Shoulder and Hand questionnaire (DASH), and Tampa Scale for Kinesiophobia-11 (TSK-11).

**Results:**

All participants completed the intervention, and baseline characteristics were comparable between groups. Compared with the control group, the dual-task group showed significantly greater improvement in CMS and greater reductions in β-ΔwPLINBS, VAS, and TSK-11. Although AAE and DASH improved over time in both groups, no significant time × group interactions were found.

**Conclusions:**

Cognitive–motor dual-task training added to conventional rehabilitation after ARCR may further improve shoulder function and reduce pain and kinesiophobia. It was also associated with changes in sensorimotor network connectivity.

*Trial registration* Chinese Clinical Trial Registry (https://www.chictr.org.cn), No. ChiCTR2400087465, 29/07/2024.

**Electronic supplementary material:**

The online version of this article (10.1186/s40798-026-01069-y) contains supplementary material, which is available to authorized users.

## Key Points

- Adding cognitive–motor dual-task training to conventional rehabilitation after arthroscopic rotator cuff repair led to better postoperative recovery, including improved shoulder function and reduced pain- and fear-related barriers to rehabilitation.
- Dual-task training was associated with a greater reduction in β-ΔwPLINBS, indicating a smaller deviation of β-band sensorimotor network connectivity from the healthy-control reference value.
- These findings support further evaluation of cognitive–motor dual-task training as an adjunct to postoperative shoulder rehabilitation, with potential benefits across clinical and EEG-based connectivity outcomes.

**Electronic supplementary material:**

The online version of this article (10.1186/s40798-026-01069-y) contains supplementary material, which is available to authorized users.

## Introduction

Rotator cuff injury is one of the most common causes of shoulder pain and functional impairment. Although arthroscopic rotator cuff repair (ARCR) can restore tendon-to-bone continuity at the anatomical level, postoperative functional recovery depends heavily on the timing, load progression, and overall quality of the rehabilitation program [1, 2]. Despite advances in surgical techniques, the postoperative immobilization and early protective phases may still be accompanied by joint stiffness, scar adhesion, pain, muscle atrophy, and delayed return to daily life or work. Therefore, achieving an optimal balance between protecting tissue healing and promoting functional recovery remains a central challenge in perioperative rehabilitation after ARCR [3]. In addition, rotator cuff injury and postoperative recovery are also influenced by impaired proprioception, altered sensorimotor control, and psychological and behavioral factors, such as kinesiophobia and pain, suggesting that rehabilitation should place greater emphasis on movement quality and the stable use of the shoulder in real-world functional contexts [4–6].

In daily life, sports, and work-related settings, upper-limb movements are rarely performed under single-task, full-attention conditions. Instead, patients are often required to manage concurrent cognitive demands, such as conversation, environmental monitoring, route planning, or simple calculations while performing motor tasks [7]. Dual-task theory proposes that when individuals perform two tasks simultaneously, competition for attentional resources and cognitive–motor interference may occur. Dual-task training (DTT), which superimposes a secondary cognitive task onto a primary motor task, is intended to improve attentional allocation, reduce interference costs, and facilitate the contextual transfer of motor control [8]. Systematic reviews and meta-analyses have shown that dual-task training can improve balance, gait, and dual-task performance in older adults and in certain neurological populations, suggesting that cognitive–motor coupling is both trainable and clinically translatable [9, 10]. In orthopedic postoperative populations, more direct clinical evidence has also emerged. For example, a study in patients following total hip arthroplasty reported that adding an 8-week dual-task intervention to conventional rehabilitation resulted in greater improvements in pain, function, dual-task performance, gait, and balance [11]. Similar findings have also been reported in the upper extremity: in elbow fracture rehabilitation, combining resistance training with a concurrent mathematical subtraction task led to greater gains in strength and greater pain reduction, and may also improve kinesiophobia [12]. Meanwhile, the sports medicine field has increasingly emphasized the need to incorporate dual-task and attentional control into shoulder rehabilitation, such as in patients with shoulder instability, to enhance contextual adaptability during return to sport and daily function. However, randomized controlled evidence regarding dual-task rehabilitation in patients following ARCR remains limited [13].

Against this background, the present study aimed to integrate a cognitive task into a standard postoperative rehabilitation program after ARCR to develop a cognitive–motor dual-task rehabilitation protocol and to evaluate its effectiveness and safety in improving postoperative shoulder function and related clinical outcomes using a randomized controlled design. We hypothesized that, compared with conventional rehabilitation alone, cognitive–motor dual-task rehabilitation would produce greater functional improvement during follow-up and would reduce the adverse impact of pain-related factors on the rehabilitation process by enhancing movement control and attentional allocation under real-world task demands.

## Methods

### Study Design and Randomization

This study was designed as a randomized, parallel-group, controlled trial and was conducted in the Department of Sports Medicine, The First Affiliated Hospital of Fujian Medical University, Fuzhou, China, between January 2025 and January 2026. The trial was prospectively registered in the Chinese Clinical Trial Registry on July 29, 2024 (ChiCTR2400087465). All eligible patients were screened in the outpatient clinic and provided written informed consent prior to participation.

**Fig. 1:**
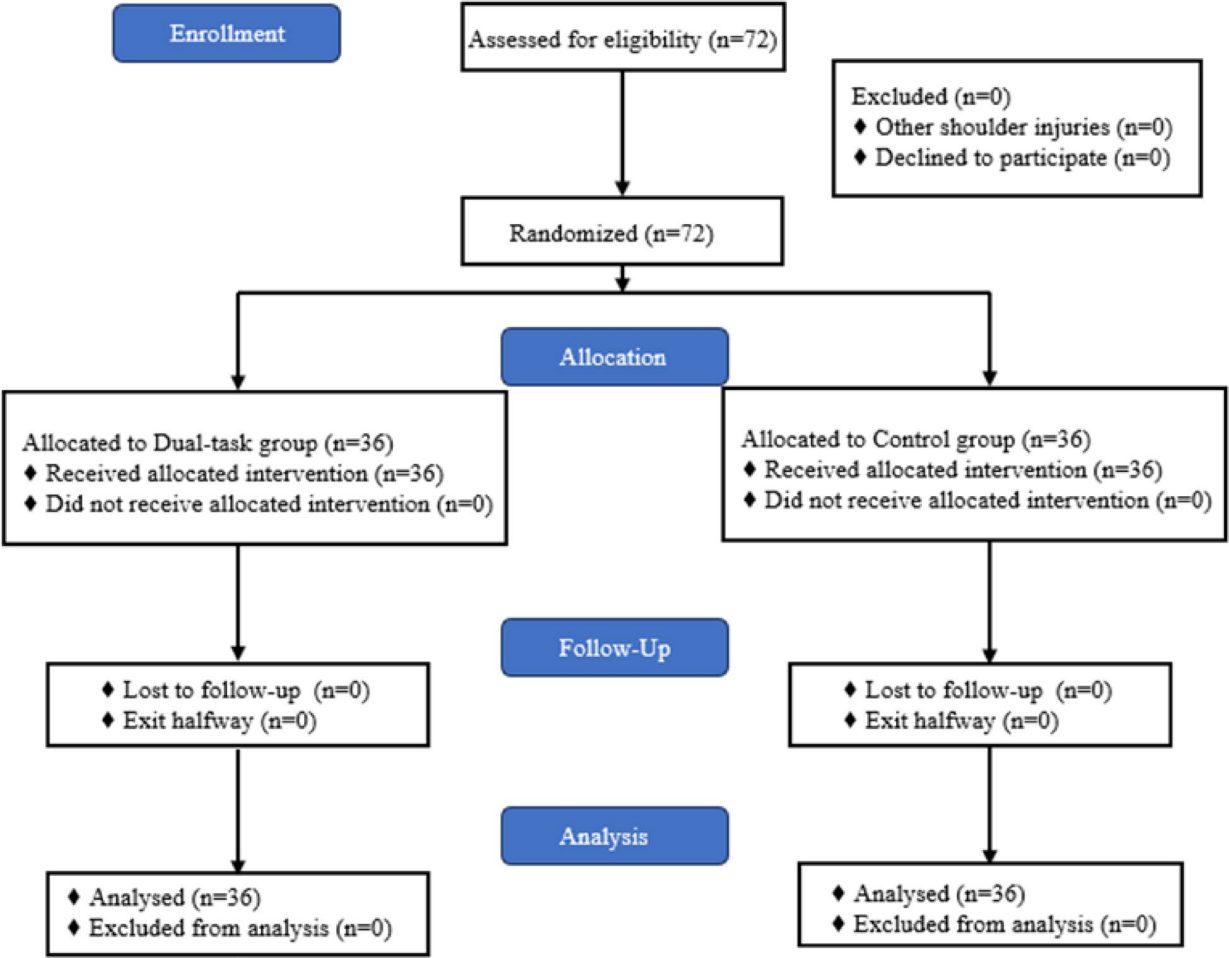
Study flowchart

Randomization was performed using a computer-generated random sequence, and participants were allocated to either the control group or the dual-task intervention group at a 1:1 ratio. Allocation concealment was ensured using sealed opaque envelopes prepared by a researcher who was not involved in the implementation of the intervention or outcome assessment. Owing to the nature of the intervention, neither the participants nor the treating therapists could be blinded. However, all outcome assessments were performed by an independent physical therapist who was blinded to group allocation (Fig. 1).

### Participants and Sample Size Calculation

The sample size was estimated using G*Power 3.1 software. Based on a 2 (group) × 2 (time) mixed-design analysis of variance, with the time × group interaction specified as the primary effect of interest, the significance level was set at α = 0.05, statistical power at 1 − β = 0.80, and the effect size at *f* = 0.30. The minimum required sample size was calculated to be 60 participants. Allowing for an anticipated dropout rate of approximately 20%, the final target sample size was set at 72 participants.

The inclusion criteria were as follows: adults with a primary full-thickness rotator cuff tear scheduled to undergo arthroscopic rotator cuff repair (ARCR), with the tear involving at least the supraspinatus tendon. The exclusion criteria were as follows: a history of previous surgery or traumatic injury to the affected shoulder; intraoperative findings indicating an irreparable rotator cuff tear; the presence of moderate-to-severe psychiatric disorders or marked social dysfunction requiring long-term pharmacological treatment or hospitalization; and failure to provide written informed consent. To reduce etiological heterogeneity, patients with traumatic tears were excluded; therefore, the cohort primarily represented non-traumatic (likely degenerative/overuse) supraspinatus-involved full-thickness tears.

### Intervention

Participants in the control group received standard perioperative care and the routine postoperative rehabilitation program. A detailed phase-specific rehabilitation protocol is provided in Supplementary Table S1. The rehabilitation protocol included wall-climbing exercises in the coronal and sagittal planes, back extension exercises, and closed-chain exercises, such as door-pushing. In the dual-task group, dual-task training was incorporated into the 12-week rehabilitation program and was delivered twice weekly under supervision.

The 12-week intervention was structured according to the postoperative healing stage and comprised an active/active-assisted phase during postoperative weeks 6–12 and a light-strengthening phase during postoperative weeks 12–18. Progression was both phase-based and criterion-based rather than determined solely by postoperative time. Specifically, participants in the dual-task group performed a self-paced serial subtraction-by-3 task concurrently with the prescribed shoulder exercises, beginning at 100 and subtracting 3 successively, without time restriction. Participants were instructed to verbalize each response aloud and to continue the subtraction task throughout the relevant exercise sets. During each supervised session, the treating therapist monitored the concurrent performance of the cognitive and motor tasks and recorded the total number of responses and the number of correct responses. Cognitive-task accuracy was calculated as the number of correct responses divided by the total number of responses and multiplied by 100%. An accuracy of ≥ 90% was used as the operational criterion for adequate cognitive-task engagement. When accuracy fell below 90%, the treating therapist repeated the task instructions and encouraged the participant to maintain attention to both the cognitive and motor tasks during the subsequent exercise set. Calculation errors were recorded but were not corrected during the ongoing exercise set to avoid interrupting concurrent task performance or providing additional cognitive cues. The subtraction-by-3 task was maintained throughout the intervention to ensure consistency of cognitive-task content across participants and sessions. Participants in the control group performed the same exercise program without the additional cognitive task.

During postoperative weeks 6–12, the cognitive task was primarily combined with active or active-assisted shoulder exercises and low-load scapular-control training. Exercises were progressed when participants were able to complete the prescribed repetitions with acceptable movement quality, appropriate scapulohumeral coordination, and without obvious compensatory movements, excessive fatigue, or sustained aggravation of shoulder symptoms. If these criteria were not met, the exercise range, number of repetitions, or exercise complexity was reduced, and further progression was postponed until adequate movement control and tolerance were achieved.

During postoperative weeks 12–18, dual-task training was further integrated into light-resistance strengthening exercises. Strengthening was progressed stepwise by first increasing the number of repetitions or sets and subsequently increasing resistance when participants could complete the prescribed exercise volume with appropriate technique and without sustained symptom aggravation. Resistance or exercise volume was reduced when compensatory movement, excessive fatigue, deterioration in movement quality, or increased shoulder discomfort was observed.

Adherence to the supervised sessions was monitored by the treating therapist based on session attendance and completion of the prescribed exercise sets. Movement quality and tolerance to the planned exercise volume were also monitored throughout each session. Exercise modifications or temporary regression of the program were determined by the treating therapist according to the same progression and safety criteria.

Outcome measures were assessed at 6 weeks postoperatively (pre-intervention) and at 18 weeks postoperatively (upon completion of the 12-week intervention). All assessments were conducted by blinded external physical therapists at the same rehabilitation facility.

### Primary Outcomes

#### The Constant–Murley Score

The Constant–Murley Score (CMS) was used to assess shoulder function. The CMS evaluates shoulder function across four domains: pain, activities of daily living (ADL), range of motion (ROM), and muscle strength. The total score ranges from 0 to 100 points, comprising 15 points for pain, 20 points for ADL, 40 points for ROM, and 25 points for muscle strength. Higher scores indicate better shoulder function.

#### β-ΔwPLINBS

**Fig. 2:**
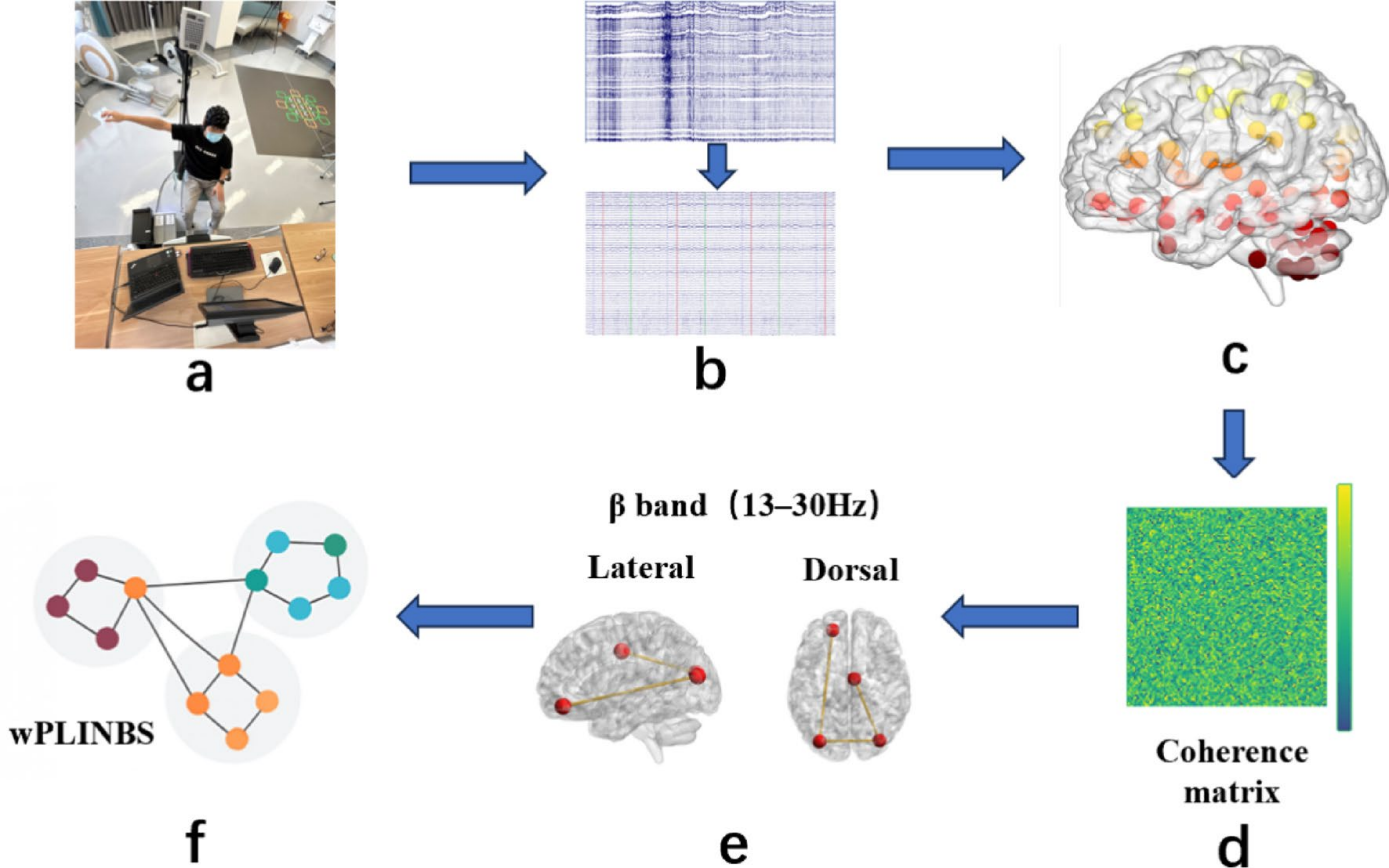
Schematic workflow of the EEG-based functional connectivity analysis. **a** EEG acquisition during the upper-limb abduction task; **b** preprocessing of raw EEG signals; **c** source localization and extraction of ROI time series; **d** construction of the β-band functional connectivity matrix based on wPLI (unitless; range 0–1); **e** identification of significant functional connectivity subnetworks using NBS; **f** extraction of the wPLINBS network metric and calculation of β-ΔwPLINBS. EEG: electroencephalography; ROI: region of interest; wPLI: weighted phase lag index; NBS: network-based statistic; wPLINBS: mean weighted phase lag index (wPLI) within the NBS-identified significant subnetwork; β: beta band; Hz: hertz; β-ΔwPLINBS: β-band weighted Phase Lag Index Network-Based Statistic difference

In this outcome measure (Fig. 2), electroencephalography (EEG) signals were recorded, while participants performed a shoulder abduction task. All participants wore a 64-channel electrode cap arranged according to the international 10–20 system. EEG data were sampled at 1024 Hz, referenced to the bilateral mastoids, and electrode impedance was maintained below 5 kΩ. During the experiment, participants were instructed to keep the head and trunk relatively stable and to perform shoulder abduction according to on-screen cues in order to minimize movement artifacts. The raw EEG data were preprocessed in MATLAB using the EEGLAB toolbox, including 0.5–80 Hz bandpass filtering, 50 Hz notch filtering, baseline correction, bad-channel detection and interpolation, common average re-referencing, and artifact removal based on independent component analysis (ICA), to obtain clean EEG time series.

Source localization was subsequently performed using minimum norm estimation (MNE), and the source signals were projected onto the AAL116 atlas to extract region-of-interest (ROI) time series for each brain region. Based on these ROI time series, the weighted phase lag index (wPLI) was calculated between brain regions to construct whole-brain functional connectivity matrices. Network-based statistic (NBS) analysis was then applied to identify functional connectivity subnetworks showing significant differences between patients after arthroscopic rotator cuff repair (ARCR) and healthy controls (HC).

The mean connectivity strength of all edges within the identified subnetwork was further calculated to derive the network mean connectivity strength index (wPLINBS). In the present study, the difference between the individual β-wPLINBS value and the mean value of the HC group was calculated to quantify the degree of brain network abnormality and was defined as β-ΔwPLINBS. A smaller β-ΔwPLINBS value indicated that the patient’s brain functional connectivity was closer to the healthy level, reflecting better recovery of the sensorimotor network. The β band was selected for analysis, because β oscillations are considered one of the most representative neural rhythms of the sensorimotor system, are closely associated with motor control and sensorimotor integration, and have been widely used in studies of motor-related brain networks [14].

### Secondary Outcomes

#### Pain

Pain intensity was assessed using the Visual Analog Scale (VAS). The VAS consists of a 100-mm horizontal line used to evaluate self-perceived pain intensity, where 0 indicates “no pain” and 100 indicates “the worst tolerable pain.” Participants were asked to mark the point on the line that best represented their current level of pain. The distance in millimeters from the left end of the scale to the marked point was then measured and recorded as the pain score.

#### AAE

Joint position sense (JPS), an important component of proprioception, refers to the ability to perceive the static spatial position of a limb in the absence of visual feedback. In the present study, absolute angular error (AAE) was used to assess neuromuscular control, as it quantifies the accuracy of reproducing a target joint angle. Previous studies have shown that AAE is a commonly used indicator for evaluating shoulder proprioceptive function, with smaller values indicating greater accuracy of joint position sense.

During testing, participants stood with their back against a wall, and an angle sensor (WT9011DCL) was secured to the wrist on the affected side. The upper limb was positioned naturally at the side as the starting position (0°), and participants wore a blindfold to eliminate visual input. The therapist then passively moved the affected upper limb to the target position (30° of shoulder abduction), maintained this position for 5 s, and returned the limb to the starting position. Participants were subsequently instructed to actively reproduce the target angle, and the actual angle achieved was recorded. Absolute angular error was calculated as follows: AAE =|actual angle − target angle|. Each participant completed three trials, and the mean value was used for statistical analysis. Smaller AAE values indicate more accurate joint position sense, whereas larger errors suggest impaired proprioceptive function [15].

#### DASH

The Disabilities of the Arm, Shoulder and Hand (DASH) questionnaire was used to assess participants’ ability to perform activities of daily living. The DASH is designed to evaluate upper-extremity functional limitation and related symptoms, with total scores ranging from 30 to 150. Higher scores indicate greater upper-extremity disability. The questionnaire has demonstrated good reliability and validity, and previous studies have reported high internal consistency [16].

#### TSK-11

The 11-item Tampa Scale for Kinesiophobia (TSK-11) was used to assess the level of fear of movement. This questionnaire consists of 11 items, each rated on a 4-point Likert scale, with total scores ranging from 11 to 44. Higher scores indicate greater kinesiophobia. Previous studies have demonstrated good reliability of the scale, with a Cronbach’s α coefficient of 0.79 [17, 18].

### Statistical Analysis

All statistical analyses were performed using Jamovi statistical software (version 2.3.24; Jamovi Project, Sydney, Australia). Continuous variables are presented as the mean ± standard deviation (SD) or mean with 95% confidence interval (CI), whereas categorical variables are presented as frequencies and percentages. Normality of data distribution was assessed using the Shapiro–Wilk test. Baseline demographic and clinical characteristics were compared between the two groups using independent-samples *t* tests or *χ*^*2*^ tests, as appropriate.

To evaluate the intervention effects, a mixed-design analysis of variance (mixed ANOVA) was performed, with time (pre-intervention vs post-intervention) as the within-subject factor and group (control group vs dual-task group) as the between-subject factor. The main effects of time and group, as well as the time × group interaction effect, were reported. When appropriate, post hoc comparisons with Bonferroni correction were conducted to examine within-group and between-group differences. This correction was applied to post hoc tests within each outcome to limit inflation of Type I error due to multiple testing. Effect sizes were expressed as ηₚ^2^, and Cohen’s d was used for between-group comparisons. All statistical tests were two-tailed, and *P* < 0.05 was considered statistically significant.

## Results

All participants completed the 12-week training program. No significant between-group differences were observed in baseline clinical characteristics or outcome measures (Table 1). During the supervised dual-task sessions, mean cognitive-task accuracy was 92.0 ± 5.6%, with session-level values ranging from 80.6% to 95.4%.

**Table 1:** Baseline demographic and clinical characteristics of the participants

	Control group (*N* = 36)	Dual-task group (*N* = 36)	*p* value
Age (years)	45.00 ± 9.00	46.50 ± 4.75	0.36
Gender (male/female)	23/13	26/10	0.45
Height (m)	1.64 ± 0.07	1.66 ± 0.08	0.11
Weight (kg)	60.23 ± 2.64	59.78 ± 4.01	0.58
Surgical side (left/right)	11/25	15/21	0.33
Dominant side involved (Yes/No)	26/10	23/13	0.45
Time since surgery (days)	43.83 ± 3.22	45.12 ± 4.19	0.15
Time from symptom onset to surgery (month)	3.63 ± 0.76	3.87 ± 0.12	0.07
Educational level (Below Bachelor’s degree/Bachelor’s degree or above)	16/20	19/17	0.48
CMS (0–100)	39.86 ± 5.70	38.36 ± 5.86	0.28
β-ΔwPLINBS	0.80 ± 0.21	0.76 ± 0.12	0.25
VAS (0–100)	46.78 ± 9.44	47.72 ± 7.32	0.64
AAE	11.16 ± 1.96	10.51 ± 2.25	0.20
DASH (30–150)	74.17 ± 6.38	73.75 ± 5.31	0.31
TSK-11 (11–44)	24.31 ± 3.65	24.72 ± 2.83	0.59

CMS: Constant–Murley Score; VAS: Visual Analog Scale; AAE: Absolute Angular Error; DASH: Disabilities of the Arm, Shoulder and Hand; TSK-11: 11-item Tampa Scale for Kinesiophobia; β-ΔwPLINBS: β-band weighted Phase Lag Index Network-Based Statistic difference

### CMS

**Fig. 3:**
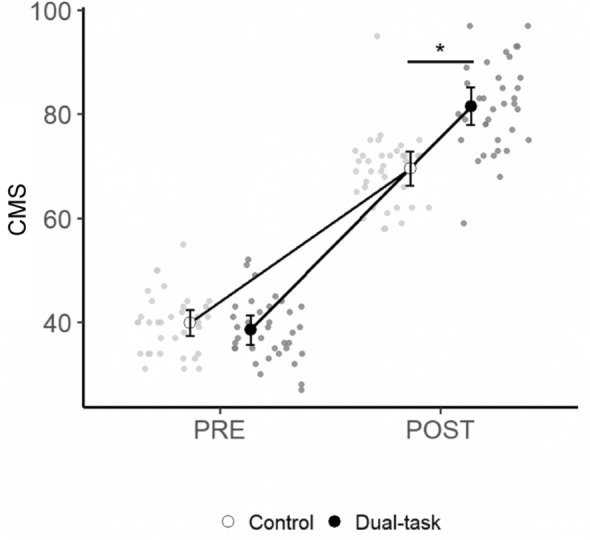
Changes in CMS before and after the intervention in the two groups. Error bars indicate 95% confidence intervals. *P* < 0.001 between groups at post-intervention. CMS: Constant–Murley Score; CI: confidence interval; Pre: postoperative week 6; Post: postoperative week 18

A significant time × group interaction was observed for CMS (*F*₍1,70₎ = 52.85, *P* < 0.001, ηₚ^2^ = 0.43). Significant main effects of time (*F*₍1,70₎ = 30.32, *P* < 0.001, ηₚ^2^ = 0.30) and group (*F*₍1,70₎ = 14.18, *P* < 0.001, ηₚ^2^ = 0.17) were also found. Post hoc analysis showed that the improvement in CMS was significantly greater in the dual-task group than in the control group (*P* < 0.001, *d* = 1.93) (Table 2, Fig. 3).

**Table 2:** Changes in clinical outcomes before and after the intervention in the two groups

		Mean	95% Confidence interval
CMS (0–100)			
Control	Pre	39.80	(37.30–42.30)
	Post	69.50	(66.30–72.80)^†^
Dual task	Pre	38.50	(35.70–41.30)
	Post	81.50	(77.90–85.20)^*‡^
β-ΔwPLINBS			
Control	Pre	0.84	(0.79–0.89)
	Post	0.33	(0.29–0.36)^†^
Dual task	Pre	0.79	(0.73–0.84)
	Post	0.13	(0.10–0.17)^*‡^
VAS (0–100)			
Control	Pre	47.60	(44.20–51.00)
	Post	22.50	(20.20–24.80)^†^
Dual task	Pre	49.80	(45.90–53.60)
	Post	10.10	(7.50–12.70)^*‡^
AAE			
Control	Pre	11.27	(10.39–12.15)
	Post	5.29	(4.79–5.80)^†^
Dual task	Pre	10.60	(9.61–11.59)
	Post	3.58	(3.01–4.14)^‡^
DASH (30–150 pts)			
Control	Pre	74.18	(71.68–76.69)
	Post	45.82	(44.15–47.49)^†^
Dual task	Pre	72.90	(70.09–75.71)
	Post	46.50	(44.63–48.37)^†^
TSK-11 (0–44 pts)			
Control	Pre	24.47	(23.32–25.62)
	Post	23.82	(22.71–24.93)
Dual task	Pre	24.83	(23.71–25.94)
	Post	19.69	(18.62–20.76)^*‡^

Pre: postoperative week 6; Post: postoperative week 18; CMS: Constant–Murley Score; VAS: Visual Analog Scale; AAE: Absolute Angular Error; DASH: Disabilities of the Arm, Shoulder and Hand; TSK-11: 11-item Tampa Scale for Kinesiophobia; β-ΔwPLINBS: β-band weighted Phase Lag Index Network-Based Statistic difference

*P < 0.001 (post, between groups)

^†^*P* < 0.001 (pre vs post within group)

^‡^*P* < 0.05 (pre vs post within group)

### β-ΔwPLINBS

**Fig. 4:**
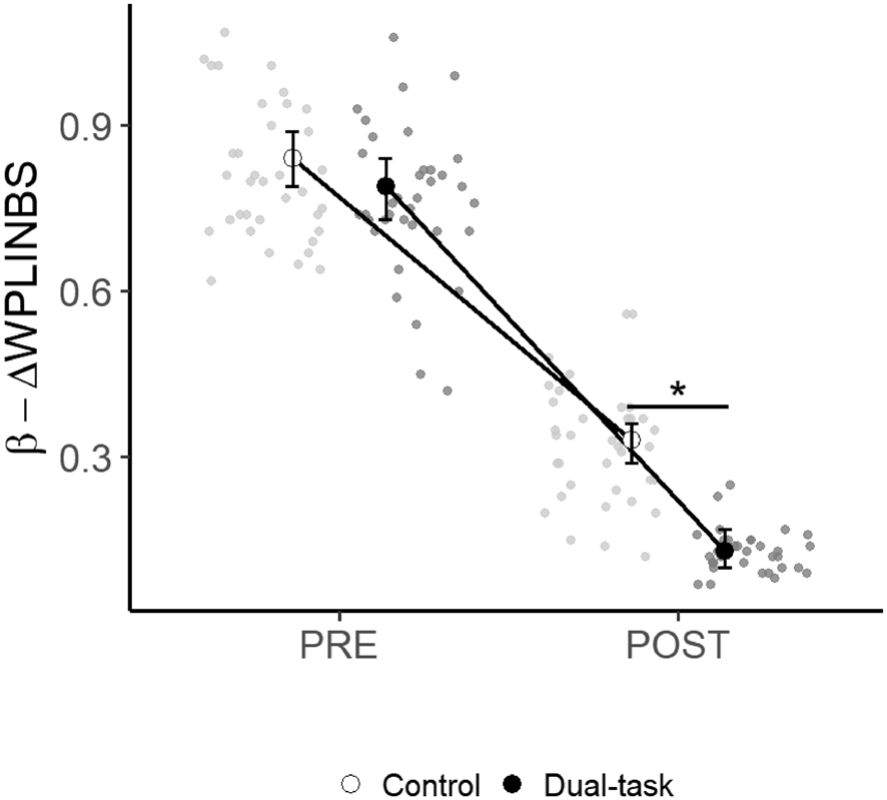
Changes in β-ΔwPLINBS before and after the intervention in the two groups. Error bars indicate 95% confidence intervals. *P* < 0.001 between groups at post-intervention. β-ΔwPLINBS, β-band weighted Phase Lag Index Network-Based Statistic difference; CI: confidence interval; Pre: postoperative week 6; Post: postoperative week 18

A significant time × group interaction was observed for β-ΔwPLINBS (*F*₍1,70₎ = 11.35, *P* = 0.001, ηₚ^2^ = 0.14). Significant main effects of time (*F*₍1,70₎ = 45.85, *P* < 0.001, ηₚ^2^ = 0.40) and group (*F*₍1,70₎ = 42.50, *P* < 0.001, ηₚ^2^ = 0.38) were also identified. Post hoc analysis demonstrated that the reduction in β-ΔwPLINBS was significantly greater in the dual-task group than in the control group (*P* < 0.001, *d* = 0.90) (Table 2, Fig. 4).

### VAS

A significant time × group interaction was found for VAS (F₍1,70₎ = 32.96, *P* < 0.001, ηₚ^2^ = 0.32). Significant main effects of time (*F*₍1,70₎ = 375.80, *P* < 0.001, ηₚ^2^ = 0.84) and group (*F*₍1,70₎ = 13.96, *P* < 0.001, ηₚ^2^ = 0.17) were also observed. Post hoc analysis showed that the reduction in VAS scores was significantly greater in the dual-task group than in the control group (*P* < 0.001, *d* = 1.56) (Table 2).

### AAE

The time × group interaction for AAE did not reach statistical significance (*F*₍1,70₎ = 2.84, *P* = 0.09, ηₚ^2^ = 0.04). However, significant main effects of time (*F*₍1,70₎ = 251.95, *P* < 0.001, ηₚ^2^ = 0.78) and group (*F*₍1,70₎ = 12.41, *P* < 0.001, ηₚ^2^ = 0.15) were observed. Post hoc analysis indicated that AAE improved significantly after the intervention in both groups, but the between-group difference in the magnitude of improvement was not statistically significant (*P* = 0.09, *d* = 0.43) (Table 2).

### DASH

The time × group interaction for DASH was not statistically significant (*F*₍1,70₎ = 1.14, *P* = 0.29, ηₚ^2^ = 0.02). A significant main effect of time was found (*F*₍1,70₎ = 311.01, *P* < 0.001, ηₚ^2^ = 0.82), whereas the main effect of group was not significant (*F*₍1,70₎ = 0.09, *P* = 0.765, ηₚ^2^ = 0.001). Post hoc analysis indicated that DASH scores improved significantly after the intervention in both groups, but the between-group difference in the magnitude of improvement was not statistically significant (*P* = 0.53, *d* = 0.13) (Table 2).

### TSK-11

Significant effects were observed for the time × group interaction (*F*₍1,70₎ = 10.53, *P* = 0.002, ηₚ^2^ = 0.13), the main effect of time (*F*₍1,70₎ = 213.55, *P* < 0.001, ηₚ^2^ = 0.75), and the main effect of group (*F*₍1,70₎ = 6.69, *P* = 0.012, ηₚ^2^ = 0.09). Post hoc analysis showed that the improvement in kinesiophobia was significantly greater in the dual-task group than in the control group (*P* < 0.001, *d* = 0.88) (Table 2).

## Discussion

The present study addressed a central question: whether the addition of cognitive–motor dual-task training after arthroscopic rotator cuff repair (ARCR) could further improve shoulder function. To address this question, we conducted a randomized, parallel group, controlled trial in which a 12-week supervised training program, delivered twice weekly, was initiated at postoperative week 6. In the dual-task group, a serial subtraction-by-3 task was superimposed on conventional shoulder rehabilitation exercises. Clinical function, pain, proprioception, kinesiophobia, and changes in the EEG-based β-ΔwPLINBS were assessed at postoperative weeks 6 and 18. The results showed that, compared with the control group, the dual-task group achieved greater improvements in CMS, VAS, TSK-11, and the β-ΔwPLINBS. By contrast, although both AAE and DASH improved over the course of rehabilitation, the between-group differences did not reach statistical significance. These findings suggest that the effects of dual-task training may be mainly reflected in shoulder function, pain reduction, psychological factors, and changes in sensorimotor network connectivity. Accordingly, the following sections discuss the consistency and discrepancies between the present findings and previous literature, several unexpected yet potentially informative observations, the relationship between these findings and relevant mechanisms, and the limitations of the present study.

The findings of the present study were largely consistent with the existing literature. First, with respect to the overall superiority of dual-task training over exercise-only rehabilitation, our results are in agreement with previous evidence from both postoperative orthopedic populations and the broader field of cognitive–motor rehabilitation. A randomized controlled trial in patients undergoing rehabilitation after elbow fracture showed that dual-task resistance training produced greater pain relief and functional improvement than resistance training alone [12]. More broadly, in the fields of sports rehabilitation and neurorehabilitation, cognitive–motor dual-task training has repeatedly been shown to improve motor performance and task adaptability under complex conditions [19]. Second, regarding recovery after ARCR itself, the overall improvements observed in pain, function, and several secondary outcomes in both groups are also in line with the established evidence base for postoperative rotator cuff rehabilitation. Systematic reviews have indicated that recovery after ARCR generally follows a progressive trajectory over time, and that outcome evaluation should encompass multiple domains, including pain, function, range of motion, and psychosocial factors [20–22]. In addition, the greater reduction in kinesiophobia observed in the dual-task group is consistent with recent evidence highlighting the importance of psychosocial factors after rotator cuff repair, suggesting that postoperative outcomes are determined not only by the structural success of the repair, but also by patients’ fear responses and patterns of engagement in rehabilitation [5, 23]. However, the present findings were not entirely concordant with previous reports, particularly in that AAE and DASH did not show significantly greater improvement in the dual-task group than in the control group. At least three explanations may account for this discrepancy. First, differences in the postoperative stage and constraints related to tissue healing may have influenced responsiveness to dual-task training. In the present study, dual-task training was initiated at postoperative week 6. Although the training content was restricted to a safe range, early rehabilitation after ARCR remains constrained by pain, protective immobilization, and tendon-to-bone healing. As a result, the additional benefits of dual-task training may not emerge simultaneously across all behavioral outcomes. Second, the observed discrepancy across outcomes may reflect differences in outcome constructs and the postoperative time window captured in this study, rather than a simple difference in “sensitivity.” AAE is a task-based measure of proprioceptive accuracy (joint position sense) at a single target angle, whereas DASH is a patient-reported measure reflecting broader upper-extremity disability and perceived limitations in complex daily activities. CMS, by contrast, is a composite score that includes symptom/function items together with clinician-assessed components such as range of motion and strength, and VAS reflects pain intensity—domains that often show substantial change during supervised rehabilitation in the early-to-mid postoperative phase. Therefore, over a 6–18-week follow-up, improvements may emerge more clearly in certain domains (e.g., pain, ROM/strength-related function, and network-level indices) than in broader patient-perceived disability or a specific proprioceptive task. The absence of a significant between-group difference in DASH may indicate that the benefits observed in clinician-assessed shoulder performance did not fully transfer to patients’ perceived ability to perform daily activities within the present follow-up period. In addition, because CMS includes performance-based components, such as range of motion and strength, it may be more responsive to supervised rehabilitation but may also be influenced by participant effort and potential performance bias related to the inability to blind participants and treating therapists, although outcome assessors were blinded. Therefore, the greater improvement in CMS should not be assumed to represent an equivalent improvement in patient-perceived daily function. Importantly, these outcomes operate at different measurement levels and should not be directly ranked against one another in terms of responsiveness [24]. Third, the primary effects of dual-task training may be more strongly directed toward central integration than toward peripheral fine joint position sense. In other words, dual-task training may initially optimize attentional allocation, sensorimotor integration, and network-level coordination, rather than immediately translating these central changes into a measurable between-group difference in joint angle reproduction accuracy.

An additional finding of the present study that was somewhat unexpected yet potentially informative was that the between-group difference in β-ΔwPLINBS emerged earlier and more clearly than that in AAE. Although AAE improved significantly in both groups, it did not demonstrate a significant additional advantage of dual-task training over the control condition. This pattern suggests that dual-task training was associated with earlier changes in sensorimotor network connectivity, with β-band connectivity showing a greater shift toward the healthy-control reference value, whereas these connectivity changes were not accompanied by a significantly greater improvement in proprioceptive accuracy. β-Band activity is widely considered to be involved in sensory integration, motor preparation, maintenance of sensorimotor states, and the utilization of contextual information. Therefore, the more pronounced reduction in β-ΔwPLINBS may reflect a smaller deviation of β-band connectivity from the healthy-control reference value [14, 25, 26]. It is also noteworthy that a significant between-group difference was observed for TSK-11, whereas DASH did not show a comparable advantage. This finding suggests that dual-task training may more readily influence psychological and control-related dimensions of recovery, such as reducing fear of movement, enhancing task engagement, and improving confidence in movement execution. However, the translation of these changes into patients’ overall subjective appraisal of limitations in complex daily activities may require a longer recovery period. Recent studies on psychosocial factors after ARCR, as well as earlier work on kinesiophobia, have shown that fear-avoidance responses are closely associated with postoperative functional recovery, although changes in these responses are not always synchronized with changes in specific functional scales [5, 23]. Importantly, the fact that not all outcome measures showed equivalent levels of sensitivity should not be regarded as a weakness. Rather, it has methodological significance, as it suggests that future studies in this field should not rely solely on a single clinical scale to evaluate the effects of dual-task training. Instead, a multidimensional outcome framework integrating clinical, psychological, and neural network measures may provide a more comprehensive understanding of the true trajectory of postoperative recovery [20, 27, 28].

To further interpret these multidomain findings, it is useful to consider dual-task theory and resource allocation models. From a theoretical perspective, simultaneous performance of cognitive and motor tasks requires individuals to allocate attention within a limited pool of processing resources. If training enhances the efficiency of this allocation, patients may achieve better movement stability, greater automaticity, and improved adaptability to environmental demands in real-life situations. In addition, changes in β-band activity and the associated network organization provide a relevant neurophysiological context for interpreting the present findings. Sensorimotor β oscillations are involved not only in motor control, but also in the integration of sensory input, prior experience, and task context. Likewise, studies of parietal–occipital–frontal networks have shown that visuospatial–motor coupling depends on dynamic coordination across distributed brain regions, and that such coordination can be reflected in β-band activity and functional connectivity [29–32]. Accordingly, the present findings suggest that rehabilitation after ARCR may involve not only improvements in strength and range of motion following peripheral tissue repair, but also changes in attentional allocation, sensory integration, and motor control. At the same time, the present findings do not support the assumption that changes in brain network connectivity necessarily correspond to uniformly greater improvements across all behavioral or sensory outcomes. The results for AAE, in particular, suggest that changes in central network connectivity, joint position sense accuracy, and complex functional performance may occur at different rates during rehabilitation. Changes in sensorimotor network connectivity, proprioceptive function, and complex activities of daily living may occur at different rates during rehabilitation; however, their temporal and clinical relationships cannot be determined from the present analyses.

## Limitations

Several limitations of the present study should be acknowledged. First, this was a single-center randomized controlled trial. Although the sample size was estimated a priori and was adequate for analysis of the primary outcomes, the statistical power may still have been limited for outcomes with greater variability or lower sensitivity, such as AAE and DASH. Second, the intervention was initiated at postoperative week 6. Although this timing is consistent with the current trend toward progressive early rehabilitation after surgery, this stage remains influenced by pain, inflammation, and protective movement restrictions; therefore, some outcomes may have reflected a combination of intervention effects, natural recovery, and ongoing tissue healing [21, 22]. Third, proprioception was assessed using AAE at a single target angle (30° abduction) in one plane, which may not fully capture angle- or plane-specific proprioceptive deficits (e.g., flexion or internal/external rotation). In addition, postoperative pain, particularly at the week-6 assessment, may have influenced AAE performance, and contralateral–shoulder comparisons were not included. Future studies should incorporate multi-angle, multi-plane testing with side-to-side comparisons and account for pain as a potential confounder. Fourth, a single serial subtraction-by-3 task was used to impose cognitive load. While this approach offered the advantages of standardization and reproducibility, it may limit the generalizability of the findings to other forms of dual-task paradigms, such as inhibitory control, visual search, or decision-making loads. Fifth, β-ΔwPLINBS is a study-specific index characterizing the deviation of β-band connectivity from the healthy-control reference value and has not been validated as a biomarker of functional recovery. It also cannot provide information regarding the directionality of connectivity changes or causal relationships. Sixth, no correlation or mediation analyses were performed between changes in β-ΔwPLINBS and changes in clinical outcomes. Therefore, the concurrent neural and clinical findings cannot be interpreted as evidence of a direct neural–clinical relationship, and this association should be examined in future studies. Seventh, although cognitive-task accuracy and concurrent cognitive–motor performance were monitored during supervised sessions, cognitive-task difficulty was not adaptively progressed and dual-task cost was not quantified. Therefore, cognitive-task accuracy primarily reflected task engagement rather than the magnitude of cognitive load or cognitive–motor interference. Future studies should incorporate adaptive task progression and single- vs dual-task assessments to quantify cognitive and motor dual-task costs. Finally, follow-up in the present study was limited to postoperative week 18. Accordingly, the long-term effects of dual-task training on functional maintenance, return to work or sports, and risk of reinjury remain unclear. Future studies with larger samples, longer follow-up periods, and more diverse dual-task paradigms are warranted, and should further incorporate mediation or path analysis to clarify the dynamic relationships among psychological factors, brain network reorganization, and clinical functional recovery.

## Conclusions

The findings of the present study indicate that incorporating cognitive–motor dual-task training into conventional rehabilitation after arthroscopic rotator cuff repair was associated with greater improvements in shoulder function, pain intensity, and kinesiophobia compared with conventional rehabilitation alone. The dual-task group also showed a greater reduction in β-ΔwPLINBS, indicating a smaller deviation of β-band functional connectivity from the healthy-control reference value. Taken together, these findings suggest that cognitive–motor dual-task training may represent a potentially valuable adjunct to postoperative rehabilitation and may be associated with changes in sensorimotor network connectivity.

## Electronic supplementary material

Below is the link to the electronic supplementary material.

Supplementary material 1 (DOCX 29 kb)

## Data Availability

The datasets used and analyzed during the current study are available from the corresponding author on reasonable request.

## Abbreviations

AAE: Absolute angular error
ARCR: Arthroscopic rotator cuff repair
CMS: Constant–Murley Shoulder Score
DASH: Disabilities of the Arm, Shoulder and Hand
HC: Healthy controls
MNE: Minimum norm estimation
NBS: Network-based statistic
ROI: Region of interest
TSK-11: 11-Item Tampa Scale for Kinesiophobia
VAS: Visual analog scale
wPLI: Weighted phase lag index
β-ΔwPLINBS: β-Band weighted Phase Lag Index Network-Based Statistic difference
