# Supplementary material for: Dual-Task Training Improves Shoulder Function and is Associated with Changes in Sensorimotor Network Connectivity After Arthroscopic Rotator Cuff Repair: A Randomized Controlled Trial
Source: Sports Med Open. 2026 Jul 13;12:96. doi: 10.1186/s40798-026-01069-y (PMC13365071; doi:10.1186/s40798-026-01069-y)
Supplement: Supplementary file 1 — Supplementary material 1 (DOCX 29 kb) [file 40798_2026_1069_MOESM1_ESM.docx]

**Supplementary Table S1. Postoperative rehabilitation protocol (weeks 6–18) and dual-task component**

| **Phase (post-op)** | **Conventional rehabilitation (both groups)** | **Dual-task addition (dual-task group)** | **Dosage & progression** |
| --- | --- | --- | --- |
| Weeks 6–12 | - ROM & control-focused - Wall-climbing (coronal/sagittal planes) - Back extension / behind-the-back reach (within tolerance) - Scapular setting/retraction - Low-load closed-chain (door-pushing) - Active-assisted → active abduction/flexion (symptom-limited) | - Serial subtraction-by-3 (start 100), self-paced during main exercise sets - No time limit or error correction - Prioritize movement quality | - 2 supervised sessions/week; ~30–45 min/session - Typically 2–3 sets × 10–15 reps per exercise - Progress when form is acceptable with pain ≤3/10 and no trunk compensation (2 consecutive sessions) |
| Weeks 12–18 | - Introduce light strengthening while maintaining ROM/control - Elastic band or light weights for abduction/external rotation - Scapular stabilizer strengthening - Progress closed-chain stability as tolerated | - Continue serial subtraction-by-3 during selected strengthening/control sets - Same instructions as weeks 6–12 | - 2 supervised sessions/week; ~30–45 min/session - Strengthening typically 2–3 sets × 8–12 reps (control/ROM 10–15 reps) - Increase load gradually with proper form and pain ≤3/10 - Avoid end-range/high-load that may compromise healing |

**Notes:**

• The dual-task intervention started at postoperative week 6 and lasted 12 weeks (weeks 6–18).

• Both groups received the same conventional rehabilitation; only the dual-task group performed the concurrent cognitive task.

• Training was supervised; sessions were modified/stopped if pain increased substantially or compensatory strategies emerged.
